# The role of fear of relapse in quality of life among patients with relapsing–remitting multiple sclerosis

**DOI:** 10.3389/fneur.2026.1770236

**Published:** 2026-03-05

**Authors:** Pınar Öztürk, Mehmet Öztürk

**Affiliations:** 1 Department of Neurology, Yenimahalle Training and Research Hospital, Ankara Yıldırım Beyazıt University, Ankara, Türkiye; 2 Department of Psychiatry, Dr. Abdurrahman Yurtaslan Ankara Oncology Training and Research Hospital, Ankara, Türkiye

**Keywords:** anxiety, fear, multiple sclerosis, quality of life, relapse

## Abstract

**Background:**

Relapsing–remitting multiple sclerosis (RRMS) is characterized by unpredictable relapses that affect neurological and psychological functioning. Among psychosocial factors, fear of relapse (FoR) has been increasingly studied in relation to quality of life (QoL). This study examined the associations of FoR and health anxiety with QoL in RRMS.

**Methods:**

In this cross-sectional study, 94 RRMS patients and 50 demographically comparable healthy controls were included. Participants completed the Expanded Disability Status Scale (EDSS), FoR Scale, Health Anxiety Inventory (HAI-18), and Short Form-36 (SF-36). Between-group comparisons were conducted for descriptive purposes. Correlation and hierarchical regression analyses were restricted to the RRMS group.

**Results:**

Compared with healthy controls, RRMS patients had higher health anxiety and lower physical and mental QoL scores (all *p* < 0.001). Within the RRMS group, FoR was negatively correlated with physical (*r* = −0.537) and mental QoL (*r* = −0.481), while health anxiety showed stronger negative correlations (all *p* < 0.001). In hierarchical regression analyses, demographic and clinical variables were not significantly associated with QoL. The inclusion of health anxiety and FoR increased explained variance to 50.4% for physical QoL and 46.8% for mental QoL. Both variables were significantly and negatively associated with QoL in final models.

**Conclusion:**

FoR and health anxiety are significantly associated with QoL in RRMS. While health anxiety demonstrated comparatively stronger associations, FoR was also related to QoL beyond demographic and clinical variables, supporting the clinical relevance of both general and disease-specific anxiety processes.

## 1 Introduction

Multiple sclerosis (MS) is a chronic immune-mediated disease characterized by inflammation and neurodegeneration in the central nervous system. It typically begins in young adulthood and affects millions of people worldwide. This disease is influenced by both genetic and environmental factors (1). The most common subtype, relapsing–remitting MS (RRMS), is seen in approximately 85% of patients and is characterized by recurrent attacks followed by periods of remission (2).

These attacks negatively affect not only physical functioning but also psychological wellbeing and social life (3). Quality of life (QoL) is a multidimensional construct influenced not only by physical health status but also by psychological wellbeing, social relationships, and coping capacities. In chronic diseases, QoL is a critical indicator not only for evaluating treatment outcomes but also for understanding how individuals can maintain their daily functioning (4).

One of the most important psychosocial factors that reduces QoL in RRMS patients is described as “fear of relapse” (FoR) (5). An unexpected relapse in RRMS patients can disrupt a person’s daily life, causing intense anxiety, depressive mood, and decreased self-esteem. Concerns about future relapses constitute an additional and persistent source of psychological stress. Each relapse can be perceived as a warning sign reminding the patient of the progressive nature of the disease and the potential risk of disability (6). Studies on chronic diseases also support this finding; it has been established that fear of disease progression is one of the main factors that profoundly affects psychological wellbeing (7). Recent studies have reported that FoR is significantly associated with QoL in RRMS patients and, in some cases, may show stronger associations than certain clinical severity indicators (8). Additionally, individuals experiencing FoR may withdraw from daily activities, experience social isolation, and develop non-compliant behaviors toward treatment; this has been associated with accelerated disability progression and declines in QoL (8). Despite the established role of clinical disability in shaping QoL, emerging evidence suggests that psychological determinants—particularly anticipatory fears related to relapse—may exert a more sustained and pervasive impact on daily functioning.

Although prior research has suggested that FoR is associated with poorer QoL in RRMS (8), existing studies remain limited in number and frequently rely on bivariate analyses or do not simultaneously account for demographic characteristics, objective disease-related indicators, and broader anxiety constructs. In particular, the relative and incremental contribution of relapse-specific fears compared with general health anxiety in shaping QoL has not been sufficiently clarified. Clarifying this distinction may improve our understanding of how disease-specific and broader anxiety processes contribute to QoL in RRMS.

Health anxiety refers to excessive worry and heightened threat perception regarding one’s current or future health status. In chronic and unpredictable conditions such as MS, elevated health anxiety may increase vigilance toward bodily sensations and amplify concerns about potential disease activity (9). While health anxiety reflects a broader and transdiagnostic pattern of health-related threat perception, FoR represents a disease-specific and context-bound anticipatory concern focused on the possibility of future MS activity (8). Examining these constructs together may clarify whether relapse-related fears represent a distinct psychological dimension or primarily reflect broader health anxiety processes influencing QoL.

In the present study, we hypothesized that FoR would be significantly associated with both the physical and mental components of QoL in patients with RRMS. Furthermore, examining FoR alongside health anxiety within a hierarchical analytical framework was expected to clarify the relative contribution of disease-specific and broader anxiety processes to QoL outcomes. Given the relatively limited number of studies examining FoR in relation to QoL in RRMS, the present study aims to contribute to the growing literature on the psychological factors influencing disease impact in this population.

## 2 Materials and methods

### 2.1 Data and cohort

This cross-sectional study included patients aged 18–65 years who presented to the Neurology outpatient clinic at Ankara Yıldırım Beyazıt University Yenimahalle Training and Research Hospital between June 15, 2025, and September 15, 2025, and were followed up as outpatients by a neurologist specializing in the field. All patients had been diagnosed with RRMS according to the 2017 revised McDonald criteria. Eligible patients had not experienced a relapse for at least 3 months, had at least a primary school education, and had no history of a persistent neurodevelopmental disorder, mild cognitive impairment, dementia, or a major psychiatric disorder (e.g., schizophrenia or bipolar disorder).

Patients who had experienced a relapse or shown signs of a relapse in the previous 3 months, did not wish to participate in the study, or did not provide consent for the study were excluded. Accordingly, 100 patients were included in the study. One patient withdrew after their data were collected and did not give permission for their data to be used, while five patients were excluded from the study due to missing data. The sample group consisted of 94 patients. *A priori* power analysis (*α* = 0.05, power = 0.80, effect size *f*^2^ = 0.15) indicated that a minimum of 50 participants was required for the regression model, confirming that sample size of 94 RRMS patients was sufficient (7).

Fifty healthy controls were recruited through convenience sampling from hospital staff and their relatives who agreed to participate. Controls were selected to have sociodemographic characteristics broadly comparable to the RRMS group (e.g., age, sex, education level, and marital status) at a group level. No individual or pairwise matching procedure was performed. Group equivalence was assessed statistically, and no significant differences were observed across the examined sociodemographic variables (Table 1). Although no statistically significant differences were observed, minor residual differences cannot be entirely excluded given the convenience-based sampling approach. The control group was included to provide contextual comparison values for health anxiety and QoL measures and was not intended to represent the general population.

**Table 1:** Distribution of demographic and clinical characteristics of patients and healthy individuals.

Variables (*n* = 144)	Total	Control (*n* = 50)	Patient (*n* = 94)	*p*-value
Age (year)	40 ± 8.7	38 ± 7.4	41 ± 9.2	0.087
Sex				0.901
Female	96 (66.7)	33 (66)	63 (67)	
Male	48 (33.3)	17 (34)	31 (33)	
Educational level				0.763
Primary school	9 (6.3)	3 (6)	6 (6.4)	
High School	49 (34)	19 (38)	30 (31.9)	
University	86 (59.7)	28 (56)	58 (61.7)	
Employment status				0.603
Employed	82 (56.9)	27 (54)	55 (58.5)	
Unemployed	62 (43.1)	23 (46)	39 (41.5)	
Marital status				0.640
Single	32 (22.2)	10 (20)	22 (23.4)	
Married	112 (77.8)	40 (80)	72 (76.6)	
Having Children				0.539
No	45 (31.3)	14 (28)	31 (33)	
Yes	99 (68.8)	36 (72)	63 (67)	
Smoking				0.784
Smoker	64 (44.4)	23 (46)	41 (43.6)	
Non-smoker	80 (55.6)	27 (54)	53 (56.4)	
Disease duration	10.9 ± 7	–	10.9 ± 7	–
Number of relapses	3 (2–5)	–	3 (2–5)	–
ARR	0.3 (0.2–0.6)	–	0.3 (0.2–0.6)	–
EDSS	1.8 ± 1.4	–	1.8 ± 1.4	–
Fear of relapse	33.1 ± 14.9	–	33.1 ± 14.9	–
HAI (main)	12.3 ± 4.5	8.7 ± 2.4	14.2 ± 4.2	<0.001
HAI (negative)	2.6 ± 1.1	2.3 ± 1.1	2.8 ± 1.1	0.024
HAI (total)	14.9 ± 4.8	11 ± 2.2	17 ± 4.6	<0.001
SF-36 (Physical)	62.1 ± 18.1	76.4 ± 6.2	54.5 ± 17.7	<0.001
SF-36 (Mental)	60.2 ± 17.2	76.2 ± 8.1	51.6 ± 14.4	<0.001

Normally distributed variables are presented as mean ± SD, non-normally distributed variables as median (IQR), and categorical variables as *n* (%).

The main focus of the present study was to examine the relationship between FoR and health related QoL within the RRMS patient group using hierarchical regression analysis. In addition, comparisons between RRMS patients and healthy controls were conducted to provide a contextual understanding of general health anxiety and QoL differences associated with MS. The FoR Scale was administered exclusively to RRMS patients, as it is a disease-specific instrument designed to assess relapse-related fears unique to individuals diagnosed with MS and is not applicable to healthy individuals. Therefore, predictive and correlational analyses were limited to the RRMS sample, whereas between-group analyses were performed for comparative purposes.

Both the patient group and healthy volunteers received verbal and written information about the study, and each participant provided informed consent. A neurologist evaluated RRMS patients and recorded their sociodemographic characteristics using the Sociodemographic Data Form. Disability level was assessed with the Expanded Disability Status Scale (EDSS), and self-report measures—the Fear of Relapse Scale, Health Anxiety Inventory (HAI-18), and SF-36—were administered. These instruments were selected because they capture both functional disability (EDSS) and psychosocial constructs relevant to relapse-related anxiety (FoR Scale, HAI-18). All assessments were administered face-to-face by a neurologist trained in the study protocol to ensure standardized data collection. Healthy controls completed the Sociodemographic Data Form, the SF-36, and HAI-18.

### 2.2 Tools

A structured sociodemographic data form was developed for the purposes of this study. The form aims to collect information about participants’ basic sociodemographic variables, such as age, gender, education level, marital status, and employment status, as well as their clinical characteristics. In this context, clinical variables such as disease duration, number of attacks, and annual attack rate (ARR) were also queried. The data obtained were used to define the demographic and clinical profile of the sample and to evaluate the relationship between the variables and psychological measurements.

The Short Form-36 Survey (SF-36) is a 36-item assessment tool developed to measure health-related QoL. It is frequently used in patients with MS. This scale provides two summary scores: the Physical Component Score and the Mental Component Score (on a scale of 0–100), in addition to eight subscales (10). The Turkish validity and reliability study was conducted by Koçyiğit et al. in 1999. The Cronbach’s alpha coefficients for each subscale of the scale ranged from 0.73 to 0.76 (11). In the present study, the summary was evaluated based on two primary subscales (physical component and mental component).

Health Anxiety Inventory (HAI-18) is a brief measure that assesses individuals’ current health-related concerns and how these concerns are reflected in their responses to potential health complaints (12). In the Turkish reliability analysis, Cronbach’s alpha internal consistency coefficient was calculated to be 0.918 (13). Each item is scored between 0 and 3, and participants select the statement that best reflects them. The total score range is between 0 and 54. High scores indicate high health anxiety. The first 14 items form the central section, while the last four items are an additional dimension related to the negative consequences of illness.

Fear of Relapse Scale is a 26-item scale developed to assess the fear of experiencing attacks in RRMS patients. Each item on the scale asks individuals to indicate the extent to which thoughts related to attack fear come to mind, and this assessment is conducted using a 5-point Likert-type scale. An increase in the scale score indicates an increase in FoR (14). The validity and reliability of the Turkish version were established by Özden et al. (15), and the scale’s internal consistency, as measured by Cronbach’s alpha coefficient was calculated to be 0.914.

### 2.3 Ethical considerations

All participants and/or their legal representatives signed a written informed consent form before participating in the study. The study was approved by the Ethics Committee of Lokman Hekim University with decision number 2025/4-1. The study was conducted in accordance with the Declaration of Helsinki and COPE guidelines.

### 2.4 Statistical analysis

The statistical analysis of the data was performed using SPSS Statistics for macOS, Version 30.0 (IBM Corp., Armonk, NY, USA). The distribution of continuous variables was assessed using the Kolmogorov–Smirnov test. Variables showing a normal distribution were summarized as mean ± standard deviation (SD); variables not normally distributed were summarized as median (interquartile range, IQR); and categorical variables were summarized as n (%). For intergroup comparisons, Student’s *t*-test was used for variables showing a normal distribution, or Welch’s test when variance homogeneity was not ensured. Categorical variables were evaluated using the chi-square or Fisher’s exact test. The healthy control group was used solely for between-group comparisons and descriptive analyses. Since disease-specific clinical variables (e.g., EDSS, ARR, number of attacks, and disease duration) were not defined in the control group, correlation and regression analyses were performed only in the MS patient group. Relationships between clinical and psychological variables in the patient group were examined using Spearman correlation analysis. Due to the non-normal distribution of some variables in the correlation analyses, the non-parametric Spearman coefficient was preferred. The correlation structure was additionally visualized using a heatmap (Supplementary material 1) to provide a graphical overview of intervariable associations. Hierarchical linear regression analysis was applied to determine the factors affecting QoL (SF-36 physical and mental component scores). The hierarchical model is structured based on conceptual proximity within a biopsychosocial framework. The first block includes background determinants such as demographic variables (age, gender, education level, employment status, smoking status), clinical variables reflecting disease burden and severity (disease duration, EDSS, ARR, number of attacks) as background determinants in the second block, and psychological variables assumed to provide additional explanatory contribution from demographic and clinical factors, Total HAI and FoR, in the third block. This approach aims to evaluate the independent contribution of psychological variables from demographic and clinical factors. Given the conceptual overlap between health anxiety and FoR, both variables were entered simultaneously in the final block to examine their incremental and independent associations with QoL outcomes while accounting for potential shared variance. The appropriateness of the linear model in regression analysis was evaluated by examining the distribution of model residuals and regression assumptions rather than the distribution of individual independent variables. In regression models, the assumptions of normality of residuals, variance homogeneity, multicollinearity, and autocorrelation assumptions were checked; variance inflation factor (VIF) values were found to be below 5, and the Durbin–Watson coefficient was approximately 2. Therefore, linear regression analysis was considered appropriate. Cases with incomplete data were excluded from the final analyses (complete-case approach). 95% confidence intervals were calculated and reported for the regression coefficients. In all analyses, *p* < 0.05 was considered statistically significant.

## 3 Results

The distribution of demographic and clinical characteristics of the patient and control groups is presented in Table 1. No statistically significant differences were observed between the groups in terms of age, gender, education level, employment status, marital status, having children, and smoking. When clinical variables were examined, HAI main, HAI negative outcome, and HAI total scores were found to be significantly higher in the patient group compared to the control group (*p* < 0.05). SF-36 physical and mental component scores for QoL were found to be significantly lower in the patient group compared to the control group (*p* < 0.05) (Table 1).

Spearman correlation analysis was conducted to examine the relationships between clinical and psychological variables in patients with RRMS. FoR showed a positive and significant correlation with HAI main (*r* = 0.329; *p* = 0.001) and HAI total (*r* = 0.318; *p* = 0.002) scores. In contrast, a statistically significant negative correlation was found between FoR and the SF-36 physical component (*r* = −0.537; *p* < 0.001) and the SF-36 mental component (*r* = −0.481; *p* < 0.001). HAI scores were also found to be negatively related to both physical and mental QoL (*r* = −0.577 to −0.610, *p* < 0.001). Furthermore, a negative correlation was found between the EDSS and the SF-36 physical component (*r* = −0.336; *p* = 0.001). A graphical heatmap representation of the correlation matrix is provided in Supplementary material 1.

Within the RRMS group, no statistically significant differences in FoR scores were observed across demographic categories (all *p* > 0.05). The groups were found to be similar in terms of gender (*p* = 0.850), educational status (*p* = 0.829), employment status (*p* = 0.924), marital status (*p* = 0.830), having children (*p* = 0.732), and smoking status (*p* = 0.19).

The results of the hierarchical regression analysis evaluating the factors affecting the SF-36 physical component score are presented in Table 2. Model 1 includes demographic variables and was found to be generally insignificant (*p* = 0.099). In Model 2, where clinical variables were added, explanatory power did not increase significantly (p = 0.099). Model 3, which included the HAI total score and FoR, became significant (*p* < 0.001) and the explanatory power of the model increased to 50.4% (Adjusted R^2^ = 0.504). Both HAI total score and FoR were significantly and negatively associated with physical QoL after adjustment for demographic and clinical variables. The standardized coefficients indicated a numerically larger association for health anxiety with physical QoL (Table 2).

**Table 2:** Hierarchical regression analysis results of factors affecting SF-36 scores in MS patients.

Model	Independent variable	SF-36 physical component ^a^					SF-36 mental component ^b^				
		*B*	Standard error	*t*	*p*-value	95% confidence interval	*B*	Standard error	*t*	*p*-value	95% confidence interval
1	Fixed	63.279	18.239	3.469	0.001	27.032 to 99.526	65.754	14.716	4.468	<0.001	36.509 to 95.000
	Age	−0.275	0.219	−1.253	0.214	−0.711 to 0.161	−0.324	0.177	−1.832	0.070	−0.676–0.028
	Gender	−5.367	4.269	−1.257	0.212	−13.851 to 3.116	−4.238	3.444	−1.231	0.222	−11.083 to 2.606
	Education status	0.180	3.266	0.055	0.956	−6.309 to 6.670	−1.979	2.635	−0.751	0.455	−7.215 to 3.257
	Employment status	−0.936	4.120	−0.227	0.821	−9.123 to 7.252	1.113	3.324	0.335	0.739	−5.493 to 7.719
	Smoking status	6.597	3.805	1.734	0.086	−0.965 to 14.159	5.210	3.070	1.697	0.093	−0.891 to 11.311
2	Fixed	61.966	19.275	3.215	0.002	23.636 to 100.29	70.919	15.579	4.552	<0.001	39.938 to 101.90
	Age	−0.241	0.265	−0.910	0.365	−0.768 to 0.286	−0.256	0.214	−1.196	0.235	−0.682 to 0.170
	Gender	−4.241	4.621	−0.918	0.361	−13.430 to 4.948	−4.616	3.735	−1.236	0.220	−12.044 to 2.811
	Education level	1.167	3.474	0.336	0.738	−5.742 to 8.076	−0.914	2.808	−0.326	0.746	−6.499 to 4.670
	Employment status	1.093	4.189	0.261	0.795	−7.237 to 9.423	1.916	3.386	0.566	0.573	−4.817 to 8.649
	Smoking status	5.770	3.835	1.505	0.136	−1.856 to 13.397	4.026	3.100	1.299	0.198	−2.138 to 10.190
	Number of attacks	−0.226	1.024	−0.220	0.826	−2.261 to 1.810	0.560	0.827	0.677	0.500	−1.085 to 2.206
	ARR	−4.704	7.423	−0.634	0.528	−19.464 to 10.057	−11.290	6.000	−1.882	0.063	−23.221 to 0.641
	Duration of illness	0.193	0.461	0.419	0.676	−0.724 to 1.111	−0.379	0.373	−1.017	0.312	−1.121 to 0.362
	EDSS	−2.637	1.941	−1.359	0.178	−6.496 to 1.223	−1.219	1.569	−0.777	0.439	−4.338 to 1.901
3	Fixed	98.234	14.644	6.708	<0.001	69.103 to 127.36	98.763	12.326	8.013	<0.001	74.244 to 123.28
	Age	−0.087	0.197	−0.443	0.659	−0.480 to 0.305	−0.129	0.166	−0.777	0.439	−0.46 to 0.201
	Gender	−1.002	3.475	−0.288	0.774	−7.914 to 5.911	−1.940	2.925	−0.663	0.509	−7.758 to 3.878
	Education level	0.818	2.531	0.323	0.747	−4.217 to 5.854	−1.197	2.131	−0.562	0.576	−5.436 to 3.042
	Employment status	−0.500	3.060	−0.163	0.871	−6.587 to 5.587	0.723	2.575	0.281	0.779	−4.4 to 5.847
	Smoking status	2.933	2.817	1.041	0.301	−2.670 to 8.537	1.879	2.371	0.793	0.430	−2.837 to 6.596
	Number of relapse	−0.276	0.746	−0.370	0.712	−1.761 to 1.209	0.530	0.628	0.843	0.402	−0.72 to 1.78
	ARR	8.068	5.673	1.422	0.159	−3.217 to 19.353	−1.662	4.775	−0.348	0.729	−11.161 to 7.837
	Duration of illness	0.089	0.342	0.261	0.795	−0.591 to 0.770	−0.474	0.288	−1.646	0.104	−1.047 to 0.099
	EDSS	−0.245	1.443	−0.170	0.866	−3.115 to 2.625	0.602	1.214	0.496	0.621	−1.814 to 3.018
	HAI (total)	−1.884	0.325	−5.792	<0.001	−2.531 to 1.237	−1.508	0.274	−5.507	<0.001	−2.052 to −0.963
	Fear of relapse	−0.487	0.102	−4.774	<0.001	−0.690 to 0.284	−0.355	0.086	−4.138	<0.001	−0.526 to −0.184

a Dependent variable: SF-36 physical component. Model 1: demographic variables (ANOVA *p* = 0.099; Adjusted *R*^2^ = 0.047); Model 2: clinical variables (ANOVA *p* = 0.099; Adjusted *R*^2^ = 0.064); Model 3: HAI (total) and FoR (ANOVA *p* < 0.001; Adjusted *R*^2^ = 0.504) were added.

b Dependent variable: SF-36 mental component. Model 1: demographic variables (ANOVA *p* = 0.058; Adjusted *R*^2^ = 0.062); Model 2: clinical variables (ANOVA *p* = 0.072; Adjusted *R*^2^ = 0.076); Model 3: HAI (total) and FoR (ANOVA *p* < 0.001; Adjusted *R*^2^ = 0.468) were added.

Categorical variables (gender: female = 1, male = 2; employment status: employed = 1, unemployed = 2; smoking status: yes = 1, no = 2; education level: elementary school = 1, high school = 2, university = 3).

In the hierarchical regression analysis evaluating factors affecting the SF-36 mental component score of QoL, demographic variables in Model 1 and clinical variables in Model 2 did not contribute significantly to the model. In Model 3, where the total HAI score and FoR were added, the model became significant (*p* < 0.001) and the explained variance increased to 46.8% (Adjusted *R*^2^ = 0.468). The HAI total score and FoR were significantly and negatively associated with mental QoL. Health anxiety and FoR were significantly associated with QoL after adjustment for clinical variables (Table 2).

## 4 Discussion

This study found that psychological factors were significantly associated with QoL in RRMS patients after controlling for demographic and clinical characteristics. Health anxiety accounted for a substantial proportion of variance in QoL, indicating a significant association with broader health-related threat perceptions. Importantly, FoR remained significantly associated with both physical and mental QoL components and contributed additional explanatory value beyond demographic and clinical variables. These findings suggest that while general health anxiety represents a transdiagnostic vulnerability, FoR remained significantly associated with QoL and may reflect a disease-specific psychological dimension within the RRMS context.

Compared with healthy controls, patients with RRMS showed significantly lower physical and mental QoL scores and significantly higher health anxiety levels. This between-group finding provides contextual support for the broader psychological burden associated with MS and is consistent with prior reports showing elevated anxiety-related symptoms and poorer health-related QoL in MS populations (16, 17).

In the present study, EDSS score, attack frequency, ARR, and health anxiety were positively and significantly correlated with FoR. Similar results have been reported in the literature. Moases Ghaffary et al. (2025) showed that clinical and psychological variables such as EDSS score, attack frequency, ARR, and health anxiety were significantly associated with FoR (18). Previous studies have suggested that relapses may worsen the disease course and accumulate over time, contributing to increased disability. Accordingly, each relapse may represent not only a transient neurological event but also a potential contributor to long-term functional decline (15, 19). It is also known that attacks can have both sudden and expectation-related effects. For many RRMS patients, experiencing a relapse may be a highly distressing experience, as it often occurs unexpectedly and abruptly disrupts the individual’s daily routine (6). These findings suggest that FoR is linked to both objective disease activity and broader anxiety patterns. At the same time, the unpredictable and disruptive nature of relapse may make FoR a more disease-specific and situationally grounded form of anxiety within the context of RRMS.

Relapse periods have also been associated with psychological distress, including increased anxiety (20). In individuals with elevated health anxiety, threat-related interpretations of bodily sensations may be associated with higher FoR, which in turn may relate to poorer self-reported QoL and potentially reduced treatment adherence (20). In particular, the uncertainty associated with relapse experiences and concerns about recurrence may contribute to a persistent state of ‘anticipatory anxiety’ in RRMS patients (6, 21). In light of our findings, like prior literature, these patterns suggest that FoR operates within broader anxiety processes while also reflecting the illness-specific uncertainty inherent to RRMS (16, 22). Although general health anxiety demonstrated a comparatively stronger association with QoL in the regression models, FoR provided additional explanatory value beyond clinical variables, supporting its role as a contextually grounded and disease-specific psychological dimension in RRMS.

This study found no significant relationship between FoR and sociodemographic variables. Similarly, a study conducted during the COVID-19 pandemic found no significant association between FoR and sociodemographic factors; with the exception of gender, as female patients reported higher FoR levels (23). While contextual and cultural influences may shape emotional responses to illness, the absence of consistent sociodemographic associations in our sample suggests that FoR is not primarily determined by stable background characteristics such as age, education, or marital status. Rather, it appears to be more closely related to illness-specific experiences and psychological processes. This interpretation is consistent with our hierarchical regression results, in which demographic variables did not significantly contribute to QoL outcomes, whereas psychological variables demonstrated greater explanatory relevance.

The fact that the FoR is independent of disease duration and age may indicate that this emotional burden is felt not only in the advanced stages of the disease but also in the early stages. Even if no significant physical disability has yet developed, patients have been found to have a lower QoL and higher levels of anxiety compared to healthy individuals (8). Taken together, these patterns indicate that FoR may reflect a cognitive–emotional process beyond immediate clinical status.

It is well established that objectively measurable factors, such as the EDSS, are associated with QoL in MS patients (24, 25). Similarly, in this study, a significant negative correlation was observed between EDSS and especially the physical component of QoL. However, no significant association was identified between the number of attacks, ARR, or disease duration and QoL within our sample. Although this differs from some previous research, these findings may suggest that disability level demonstrates a clearer association with QoL than other clinical indicators within this cross-sectional context (8, 26). Importantly, this should not be interpreted as suggesting that broader clinical burden is unimportant in MS, as disability remains an established contributor to functional outcomes.

Furthermore, we cannot explain an individual’s QoL solely in terms of external conditions or objective indicators. Objective parameters undoubtedly influence wellbeing; however, they may not fully capture patients’ lived experience and subjective appraisal of their condition (8). In the hierarchical regression analysis, the inclusion of both the HAI total score and FoR in the final model was associated with a notable increase in explained variance for both physical and mental QoL components. After adjustment for sociodemographic and clinical variables, both health anxiety and FoR showed significant negative associations with QoL scores. Although health anxiety accounted for a larger proportion of variance, FoR remained significantly associated with QoL when considered alongside general health anxiety. These findings suggest that relapse-related fears are meaningfully associated with patients’ perceived wellbeing within the broader context of anxiety processes (27). Consistent with our findings, Broche-Perez et al. (5) also reported FoR as a significant correlate of QoL in MS patients.

From a clinical perspective, these findings suggest that, alongside neurological parameters, relapse-related fears may warrant attention in routine MS care. Elevated FoR levels may reflect maladaptive interpretations of bodily sensations and uncertainty regarding disease progression (28). In this context, structured psychosocial approaches such as CBT, mindfulness-based strategies, or psychoeducation have been proposed in the literature as supportive interventions targeting catastrophic expectations (14, 29), intolerance of uncertainty, and avoidance behaviors (30). Although the present study did not evaluate treatment outcomes, awareness of these psychological mechanisms may assist clinicians in adopting a more comprehensive and patient-centered approach to MS management.

There are limited studies on this subject in the literature (8). To the best of our knowledge, no prior study conducted in our country has specifically examined the relationship between FoR and QoL in patients with RRMS. Although anxiety and QoL have been examined in MS populations, studies specifically addressing FoR remain scarce. Moreover, the combined evaluation of FoR, clinical disability, and general health anxiety within the same multivariate model is limited (5, 8). The present study addresses the relatively recent FoR construct and examines it together with objective clinical variables such as EDSS and with health anxiety within a QoL framework. In addition, the sample size of the current study is larger than that reported in several previous studies examining similar constructs.

This study has several limitations. It was conducted at a single center and included participants from the same country, which may restrict the generalizability of the findings. Additionally, the healthy control group was recruited from hospital staff and their relatives, which may have introduced selection bias related to differences in higher health literacy and health-seeking behaviors compared with the general population. The cross-sectional design precludes causal inference and does not allow determination of the temporal relationship between psychological factors and QoL outcomes. Although all patients were under routine disease-modifying treatment, detailed data on medication subtypes and treatment duration were not systematically recorded for research purposes. Consequently, treatment-related effects could not be included in the analytical models. Future multicenter and longitudinal studies incorporating standardized treatment variables, fatigue, and cognitive functioning are needed to clarify these associations.

## 5 Conclusion

This study found that FoR was significantly associated with both the physical and mental components of QoL in patients with RRMS. The findings suggest that FoR appears to function as a clinically meaningful, disease-specific psychological dimension associated with QoL in RRMS, even when considered alongside broader health anxiety processes. Although health anxiety demonstrated comparatively greater associations with QoL, relapse-specific fears provided additional explanatory value beyond clinical disease burden, underscoring the importance of addressing both general and disease-specific anxiety processes in RRMS care. From a clinical perspective, these findings highlight the potential value of assessing FoR within routine MS care. In individuals reporting higher levels of FoR, structured psychosocial interventions such as psychoeducation, CBT, or mindfulness-based approaches may be considered as supportive strategies aimed at improving psychological adaptation and overall wellbeing.

In addition, incorporating validated FoR screening tools into clinical evaluation may facilitate the identification of patients experiencing elevated relapse-related concerns and support a more individualized, multidisciplinary approach to care. Such an approach aligns with biopsychosocial models of MS management and may contribute to a more comprehensive understanding of patient wellbeing.

## Data Availability

The datasets presented in this article are not readily available because the datasets generated and/or analyzed during the current study are not publicly available due to ethical and privacy considerations related to sensitive clinical data but are available from the corresponding author upon reasonable request and following acceptance of the manuscript. Requests to access the datasets should be directed to pnr_akyz@hotmail.com.
