# Supplementary material for: The role of fear of relapse in quality of life among patients with relapsing–remitting multiple sclerosis
Source: Front Neurol. 2026 Mar 5;17:1770236. doi: 10.3389/fneur.2026.1770236 (PMC12999871; doi:10.3389/fneur.2026.1770236)
Supplement: Supplementary file 1 [file Table_1.DOCX]

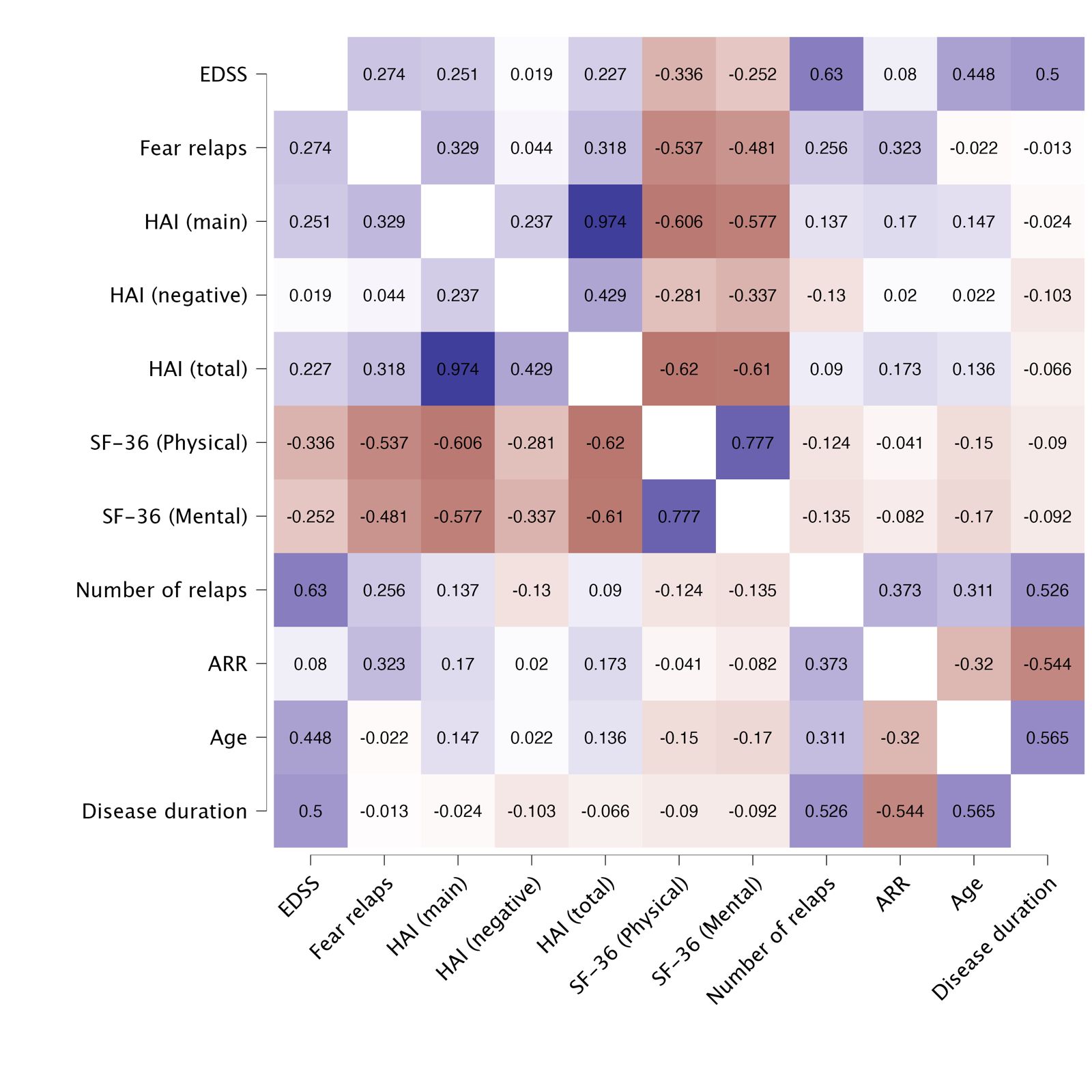


**Supplementary Material 1.** Heatmap of correlations between demographic, clinical and psychological variables in MS patients (Spearman’s correlation analysis; r =Correlation coefficient)
